# Digitalisierung christlicher Verkündigungsformate während der Covid19-Pandemie: Empirische Befunde und theoretische Systematisierungen aus religionswissenschaftlicher Perspektive

**DOI:** 10.1007/s41682-023-00149-0

**Published:** 2023-03-29

**Authors:** Anna Neumaier

**Affiliations:** Centrum für Religionswissenschaftliche Studien, Ruhr-Universität Bochum, Bochum, Deutschland

**Keywords:** Religion, Digitalisierung, Medien, Internet, Corona, Religion, Digital media, Internet, Covid, Religious authority, Religious community

## Abstract

Mit den Kontaktbeschränkungen zu Beginn der Covid19-Pandemie mussten auch religiöse Institutionen geradezu über Nacht ihre bisherigen Angebote neu denken. Gerade für Gottesdienste sind dabei häufig digitale Alternativen entwickelt worden. Der Aufsatz beleuchtet dieses emergierende Feld der Digitalisierung christlicher Verkündigungsformate und die Anschlussstellen zu bestehender religionswissenschaftlicher Forschung zu Religion und digitalen Medien. Dafür wird zunächst ein empirisch orientierter Überblick über die Digitalisierungsbemühungen der christlichen Kirchen im deutschsprachigen Raum unter Corona-Bedingungen gegeben, insbesondere unter Rückgriff auf die bisher vorliegenden statistischen Erhebungen. Auf der Grundlage qualitativer Interviews wird dann der Blick auf die Rezipient*innenperspektive auf digitale Gottesdienste gerichtet. Diese empirischen Befunde werden schließlich in Verbindung gesetzt zu bestehenden systematischen Querschnittsthemen der Forschung zu Religion und Medien, insbesondere zu den Konsequenzen digitalisierter religiöser Kommunikation für Formen religiöser Vergemeinschaftung, Raum und Ritual sowie die Selbstermächtigung religiöser Lai*innen und den Wandel religiöser Autorität. Der Aufsatz will damit auf empirischer Grundlage eine Einordnung der gegenwärtigen, Corona-induzierten Entwicklungen in den bestehenden Forschungsstand anbieten und Anschlussüberlegungen aufzeigen.

## Abstract (en)

With the contact restrictions at the beginning of the Covid19 pandemic, religious institutions also had to rethink their previous services almost overnight. Especially in the case of worships, mainly digital alternatives emerged. This article sheds light on this evolving field of the digitalisation of Christian worship formats and its junctions to existing religious studies research on religion and digital media. As a starting point, it provides an empirical overview of the digitisation efforts of Christian churches in German-speaking countries under Covid19 conditions, drawing on the statistical surveys available so far. On the basis of qualitative interviews, then, the recipients’ perspective on digital services is addressed. Lastly, these empirical findings are discussed in relation to the already debated systematic topics within research on religion and media, specifically the consequences of digitalised religious communication for forms of religious community, space and ritual as well as the self-empowerment of religious laypersons and the challenge of religious authority. The paper thus aims to offer some empirically grounded, preliminary considerations on the current covid-induced developments in the field of (Christian) religion and digital media, to integrate them into the existing state of research and to point out follow-up considerations.

## Einleitung

„Die Kirchen werden erfinderisch“, so titelte die Tagesschau auf ihrer Webseite am 10. April 2020. Mitte März 2020 wurden aufgrund der ersten Fälle von Covid19-Erkrankungen in Deutschland weitreichende Kontaktbeschränkungen beschlossen.^1^ Sie betrafen auch die Angebote religiöser Gemeinschaften, und kamen aus kirchlicher Sicht vielleicht zu einem besonders herausfordernden Zeitpunkt: Nur drei Wochen später standen die Osterfeiertage an. Beinahe über Nacht – und unter der Ungewissheit der weiteren pandemischen Entwicklungen – mussten alternative Angebote zu den traditionellen Gottesdiensten in Kirchen und Gemeindehäusern entwickelt werden.

Diese Umstellung betraf gleichwohl nicht nur die christlichen Gemeinschaften, sondern auch alle anderen religiösen Traditionen, deren Praxis gemeinschaftliche Zusammenkünfte beinhaltet. Gerade in jüdischen und muslimischen Traditionen fanden im Jahr 2020 beinahe zeitgleich Pessach und der Beginn des Ramadans statt, und damit zentrale religiöse Feiern, die ähnliche Herausforderungen für Religionsgemeinschaften wie das Osterfest mit sich brachten. In all diesen Traditionen wurden ganz verschiedene Lösungen gefunden, um mit den staatlich verordneten Kontaktbeschränkungen umzugehen. Dabei wurden die traditionellen Formate religiöser Praxis nicht in jedem Fall ins Digitale verlagert, sondern teilweise auch einfach aus geschlossenen Räumen herausverlegt: So trafen sich Muslime in Wetzlar zum Open-Air-Gebet auf einem Ikea-Parkplatz^2^, in verschiedenen christlichen Gemeinden wurden Materialien zur Abholung bereit gestellt oder Stationenwege eingerichtet, die zusätzlich zum Transfer unter offenem Himmel auch durch die Verstreuung der Teilnehmenden der Infektionsgefahr begegnen sollten.^3^ Aus der Not geboren zeigten sich vielerorts Kreativität und Experimentierfreude, die seitens der Verantwortlichen zeitgleich mit ethischen Bedenken, theologischen Erwägungen und technischen Herausforderungen abgewogen werden mussten.

Unter all diesen Umgestaltungen religiöser Praxis unter Corona-Bedingungen fokussiert der vorliegende Aufsatz ausschließlich die Übertragung traditioneller religiöser – und in diesem Fall christlicher – kollektiver Praktiken ins Digitale. Dies ist zum Einen als empirischer Fall aufschlussreich: Die Digitalisierung religiöser Kommunikation und religiöser Praxis ist in den letzten Jahren zu einem religionswissenschaftlich und -soziologisch hochrelevanten Thema geworden, das nicht zuletzt durch den sich weiterhin vollziehenden rasanten Medienwandel unaufhaltsam neue Fragen aufwirft.^4^ Der Fall der Digitalisierung religiöser Formate unter Corona-Bedingungen weist aber zum Anderen auch über sich hinaus, und zwar auf mindestens drei Arten: Er spiegelt zurück auf nicht-digitale religiöse Praxis, die sich mit dem Aufkommen der digitalen Alternativen neuen Fragen und Erprobungen stellen muss; er erhellt zentrale systematische und komparative Fragen religiöser Transformationsprozesse etwa im Bereich religiöser Gemeinschaftlichkeit, religiöser Autorität oder Ritualpraxis; und er verweist auf religionshistorische Dynamiken im Wandel von Medien- und Religionsgeschichte.

Der vorliegende Aufsatz adressiert aus dieser thematischen Bandbreite aus dem Feld von Corona, Kirche und Digitalisierung drei ineinander übergehende Fragen: Zunächst, wie traditionelle christlich-kirchliche Angebote anlässlich der Corona-Pandemie ins Digitale transferiert wurden, dann, welche Erkenntnisse bisher über die Rezeption dieser Angebote vorliegen, und schließlich, wie sich diese empirischen Erhebungen an größere theoretische und systematische Ansätze im Feld religionswissenschaftlicher Medienforschung anschließen lassen. Der Aufbau des Textes folgt dafür einem entsprechenden Dreischritt: Zunächst wird ein Überblick gegeben über die Digitalisierungsbemühungen der christlichen Kirchen im deutschsprachigen Raum unter Corona-Bedingungen, anschließend soll der Blick auf die Perspektive der Rezipient*innen und Teilnehmer*innen an den digitalisierten Gottesdienst- und Andachtsformaten gerichtet werden. Zu diesen beiden Bereichen liegen bereits erste empirische Daten vor, auf die sich die Erörterungen stützen. Zuletzt werden dieses noch junge Phänomen und die zugehörigen empirischen Befunde in Verbindung gesetzt zu bestehenden systematischen Querschnittsthemen der religionswissenschaftlichen Forschung zu Religion und Medien, insbesondere zu den Konsequenzen digitalisierter religiöser Kommunikation für Formen religiöser Vergemeinschaftung, Raum und Ritual sowie die Selbstermächtigung religiöser Lai*innen und den Wandel religiöser Autorität. Der Aufsatz will damit eine Ordnung dieses neuen Forschungsfeldes der gegenwärtigen, Covid19-induzierten Entwicklungen hinsichtlich größerer theoretischer Zugänge aus der religionswissenschaftlichen Medienforschung anbieten, und im Hinblick darauf erste Schlüsse aus den bisher vorliegenden empirischen Daten ziehen.

Was hier im Folgenden außen vor bleiben muss, und deshalb nur eingangs kurz aufgerufen werden soll, um das Bild zu vervollständigen, ist der Blick auf die innerkirchlichen und -theologischen Aushandlungen rund um den Transfer religiöser Praxis (vgl. u. a. van Oorschot 2020; Beck et al. 2021; Beck 2021; Deeg 2021a; Grethlein 2021; Winter 2020). Insbesondere die Übertragung sakramentaler Handlungen wie der Eucharistie-Feier ins Digitale stand im Zentrum einiger Diskussionen seit Pandemiebeginn und wies zudem spannende Querverbindungen zu den parallel stattfindenden Aushandlungsprozessen anderer religiöser Traditionen auf, etwa die Debatte um den Technikgebrauch unter orthodoxen Jüd*innen im Angesicht von digitalen Sederfeiern. Auch diese Fragen treten als grundsätzliche Thematik nicht zum ersten Mal auf: Wie Arbeiten zum Ritualtransfer zeigen, bedeutet ein sich wandelnder gesellschaftlicher Kontext immer auch Anfragen an die Durchführung religiöser Praxis^5^, und gerade in Bezug auf Mediennutzung gibt es dazu bereits vielfältige Auseinandersetzungen – etwa in den Debatten um die Einführung der Fernsehgottesdienste, die aufschlussreiche Parallelen aufweisen.^6^ Durch den Umstand, dass sich hier immer wieder Fragen von medialen Charakteristika sowie religiöser Medienaneignung mit dogmatischen oder liturgischen Erörterungen kreuzen, entstehen einerseits die oben genannten Rückfragen an medienunabhängige theologische Grundsätze, andererseits Diskurse um die religiöse Legitimation von Technik- bzw. Mediennutzung innerhalb mehr oder weniger medienaffinen Religionsgemeinschaften. Dieses Feld ist auch aus religionswissenschaftlicher Perspektive an anderer Stelle bereits beleuchtet worden (vgl. grundlegend Campbell 2010a).

## Die Digitalisierung kirchlicher Angebote anlässlich der Corona-Pandemie

Mit den Kontaktbeschränkungen der beginnenden Corona-Pandemie wurden im Feld christlicher Traditionen vor allem die Gottesdienste zum herausfordernden Format. Erstens zeichnen sie sich im Vergleich mit anderen kirchlichen Formaten (wie seelsorgerlichen Angeboten oder kleineren Gruppen) durch die größte Teilnehmerzahl aus, weshalb sie zunächst unter das Verbot der strikten Kontaktbeschränkungen fielen, und auch nach dessen Aufhebung seitens der Religionsgemeinschaften teilweise nicht oder unter größter Vorsicht und veränderten Rahmenbedingungen begangen wurden.^7^ Die Herausforderung betrifft zweitens räumliche Anordnung und Abläufe: Gottesdienste finden im Regelfall in geschlossenen Räumen statt, je nach Zahl der Teilnehmer*innen und baulicher Gegebenheit in enger Bestuhlung, und integrieren mit dem gemeinsamen Singen eine besonders aerosolemittierende kollektive Praxis. Drittens gelten sie aber als besonders essentielle Praxis im Leben einer Gemeinde, sowohl aus theologischer als auch sozialpsychologischer Sicht. Damit war im Frühling 2020 schnell klar, dass es unumgänglich ist, auch unter den vorgegebenen Kontaktbeschränkungen einen Ersatz zumindest für die sonntäglichen Gottesdienste zu finden. Ohne die kirchlichen Diskurse hierzu nachzeichnen zu können, ist es gleichwohl bemerkenswert, dass an dieser Stelle eben nicht auf die bereits etablierten Formate der Fernseh- und Radiogottesdienste verwiesen wurde. Vielmehr entschied sich ein großer Teil der Gemeinden, den jeweils lokalen Gottesdienst ins Digitale zu transferieren. Damit kristallisierte sich nicht nur das liturgische Format, sondern auch das Kollektiv der Gemeinde vor Ort als Maßgabe für die Entwicklung von Alternativen heraus.

Im Laufe des Jahres 2020 wurden die ersten größeren Datenerhebungen zu diesen digitalisierten Gottesdiensten und verwandten Formaten durchgeführt. Hier sind insbesondere zu nennen die Erhebung des midi-Institutes zu digitalen Verkündigungsformaten (Hörsch 2020; im Folgenden auch „Midi-Studie“), die Studie „Churches Online in Times of Corona“ (s. für einen ersten Einblick Schlag 2022; im Folgenden auch „Contoc-Studie“), die Studie der Initiative „Gott@digital“ (Wehrstein und Zettl 2020; im Folgenden auch „Digital-Studie“) und die Studie „Rezipiententypologie evangelischer Online-Gottesdienstbesucher*innen während und nach der Corona-Krise“ (Arndt et al. 2020; im Folgenden auch „ReTeoG-Studie“). Dabei wurde zur Midi-Studie ebenso wie zur ReTeoG-Studie jeweils 2021 noch eine Follow-Up-Befragung durchgeführt (Hörsch 2021; Reimann und Sievert 2021). Drei dieser Studien (Midi-Studie, Contoc-Studie, Digital-Studie) haben vor allem grundlegende Daten und Zahlen rund um die seit dem Frühjahr 2020 initiierten Online-Gottesdienste erhoben sowie die Einschätzung aus der Gruppe der Hauptamtlichen und Ehrenamtlichen in den Kirchen, die den Umstieg primär verantwortet oder begleitet haben. Diese drei Studien werden in diesem Kapitel im Mittelpunkt stehen. Die ReTeoG-Studie sowie in kleineren Teilen auch die Digital-Studie blicken auf die Sicht der Teilnehmenden an den Digitalformaten und werden daher im folgenden Kapitel erörtert.

Bei diesen Studien handelt es sich um unterschiedlich groß angelegte und nichtrepräsentative Studien, ihre Datenerhebungen und Auswertungen genügen in unterschiedlichem Maße methodischen Standards quantitativer Forschung. Die Ergebnisse bedürfen also einer entsprechend vorsichtigen Ausdeutung, sind allerdings derzeit überhaupt die einzigen vorliegenden quantitativen Daten zu diesem Forschungsbereich im deutschsprachigen Raum und können so zumindest einen näherungsweisen Blick über das Feld geben. Nötig ist daher aber eine kurze Vorstellung der Datengrundlagen: Die Contoc-Studie ist eine großangelegte internationale Studie zu den Erfahrungen von Seelsorger*innen und Pfarrer*innen im Kontext der Corona-Pandemie und den Digitalisierungsbemühungen in diesem Zuge. Hinter der Studie steht ein internationales und ökumenisches Forschungskonsortium.^8^ Die erste Datenerhebung fand in den Sommermonaten 2020 statt, bisher sind nur Ausschnitte aus den Befunden veröffentlich worden.^9^ Ein Nachfolgeprojekt, „Contoc^2^“^10^, richtet sich auf die Nutzung digitaler Medien in evangelischen Kirchengemeinden in Deutschland und der Schweiz seit Ausbruch der Corona-Pandemie, hierzu liegen aber noch keine Auswertungen vor. Hinter der „Digitalstudie“ steht ein vereinsförmiges, interkonfessionelles Netzwerk, dass sich „digitalen Möglichkeiten für Christen aus allen Konfessionen“ (Wehrstein und Zettl 2020) widmet. Zur Teilnahme an der Studie wurden haupt- und ehrenamtliche Mitarbeiter*innen in den Gemeinden über die Kanäle der Initiative eingeladen, insgesamt 442 Datensätze liegen vor. Die Befragung richtete sich grundsätzlich an das deutschsprachige, christliche Spektrum, die vorliegenden Daten und Kanäle für Teilnahmeaufrufe legen aber nahe, dass ein Schwerpunkt der Datensätze im evangelisch-landeskirchlichen sowie -freikirchlichen Spektrum zu verorten ist (vgl. ebd., S. 5). Die Midi-Studie (Hörsch 2020) wurde durchgeführt durch die Evangelische Arbeitsstelle midi, und richtet sich auf digitale Verkündigungsangebote (Gottesdienste, Andachten und andachtsähnliche Formate). Die Datenerhebung fand im Mai 2020 statt, kontaktiert wurden 116 Kirchenkreise/Dekanate aus vier evangelischen Landeskirchen^11^ mit der Bitte auch um Weiterleitung an evangelische Werke, Dienste und weitere Einrichtungen (vgl. ebd., S. 16 f.). Die im Sinne der Studienfrage verwertbaren Datensätze umfassen 729 Rückmeldungen. Eine Follow-Up-Studie im Folgejahr (Hörsch 2021) inkludierte zusätzliche weitere kirchliche Arbeitsfelder (wie Seelsorge und die Arbeit mit verschiedenen spezifischen Zielgruppen), wurde im Juni 2021 durchgeführt und bezog sich auf alle vorangegangenen Phasen der Pandemie (vgl. ebd., S. 13 f.). Adressiert wurden die Respondent*innen der ersten Erhebung, die während Corona digitale Verkündigungsformate angeboten hatten, aus diesem Sample gingen 194 Rückmeldungen ein (vgl. ebd., S. 17).

Insgesamt muss damit festgehalten werden, dass das evangelisch-landeskirchliche Feld nicht ausschließlich das Sample ausmacht, wohl aber einen Schwerpunkt in den bisher vorliegenden Daten.^12^ Darüber hinaus muss für die Interpretation berücksichtigt werden, dass die vorliegenden Studien damit auf wechselnde Pandemiephasen zurückblicken, in denen analoge Gottesdienste teilweise untersagt, teilweise unter verschiedenen Hygieneauflagen gestattet waren. Während diese Phasen zumindest teilweise von den Studien differenziert betrachtet werden (vgl. Hörsch 2021), muss zusätzlich mitgedacht werden, dass gleichzeitig auch die religiösen Anbieter verschiedene Haltungen zur Durchführung analoger Gottesdienste entwickelten, mithin das parallel stattfindende analoge Gottesdienstangebot sich bei den Befragten vor Ort jeweils sehr unterschiedlich gestaltete.

Zunächst ein allgemeiner Blick auf die Digitalisierungsbemühungen kirchlicher Angebote zu Beginn der Pandemie insgesamt: Die erste Midi-Studie zeigt, dass gut drei Viertel der Befragten während der ersten Corona-Monate ein digitales Gottesdienstangebot produziert haben, knapp 70 % haben darüber hinaus auch Andachten und 44 % andachtsähnliche Formate in digitaler Form bereitgestellt (vgl. Hörsch 2020, S. 22). Der ganz überwiegende Teil dieser Angebote entstand dabei tatsächlich ausgelöst von den Bedingungen der Corona-Pandemie und in Konstellationen, in denen vor Corona keine digitalen Verkündigungsformate angeboten wurden (vgl. Hörsch 2021; auch Wehrstein und Zettl 2020, S. 5). Die Verfasser der Midi-Studie deuten dies als „Digitalisierungsschub“ in der evangelischen Kirche (vgl. Hörsch 2021, S. 21): „Waren es vor der Covid19-Pandemie vermutlich so genannte ‚Pioniere‘, die sich auf den Weg der Digitalisierung gemacht hatten, so wurde aus der Not der Corona-Krise heraus eine disruptive digitale Transformation in der Breite der jeweiligen Landeskirchen vollzogen“ (ebd., S. 22).

Während sich die Ergebnisse der ersten Midi-Studie auf Verkündigungsformate im engeren Sinne – präzise: Gottesdienste, Andachten und sogenannte „andachtsähnliche Formate“ – beziehen, inkludiert die Digitalstudie eine größere Bandbreite aller digitalisierten kirchlichen Angebote in ihre Erhebung. Doch auch hier zeigt sich, dass der Gottesdienst die mit Abstand meistdigitalisierte und auch als solche genutzte Gattung war, gefolgt von seelsorgerlichen Angeboten, Hauskreisen, Jugendgruppen, Gebetsgruppen und Andachten (vgl. Wehrstein und Zettl 2020, S. 6). Die zweite Midi-Erhebung blickt dann auch auf seelsorgerliche Angebote, zeigt aber, dass diese im Vergleich zu den Gottesdiensten aus der Einschätzung der Anbieter nicht gut angenommen wurden (vgl. Hörsch 2021, S. 36).^13^ Auch die Digital-Studie zeigt, dass mit großem Abstand vor allem der Gottesdienst online nachgefragt wurde, weiterhin, aber schon deutlich geringer Hauskreise, Andachten, Gebetsgruppen und sonstige Gesprächskreise (vgl. Wehrstein und Zettl 2020, S. 5). Insgesamt lassen sich also breite Digitalisierungsbemühungen verschiedener Formate verzeichnen, aber mit unterschiedlicher Nachfrageintensität. Dass der Gottesdienst das Format mit der größten Nachfrage ist, mag seinen Grund ebenso darin haben, dass er in der Wahrnehmung mancher eher als andere – etwa seelsorgerliche – Formate online replizierbar ist wie darin, dass er auch offline möglicherweise einfach das gefragteste kirchliche Angebot darstellt.

Dennoch: Im Vergleich der Reichweite der digitalen Gottesdienste mit den durchschnittlichen Besucherzahlen eines Sonntagsgottesdienstes verweist die Midi-Studie auf ein Plus von 287 % im Fall der digitalen Angebote (vgl. Hörsch 2020, S. 31). Das legt nahe, dass hier neue Zielgruppen erschlossen werden konnten, aber auch, dass es zudem gelungen sei, „die Stammklientel der Verkündigung, die klassische Gottesdienstgemeinde am Sonntagmorgen mitzunehmen auf dem Weg in die Digitalität“ (ebd., S. 48).^14^ Dabei müssen diese Angaben natürlich mit Vorsicht betrachtet werden, da die Berechnungen eben nur mittelbar über die Angaben der Verantwortlichen in den Landeskirchen erhoben wurden und Faktoren wie die Verweildauer nach dem Anklicken der digitalen Angebote außen vor lassen. Auch die Digitalstudie aber zeigt hohe Zustimmungswerte zu den Aussagen, dass mehr Menschen als zuvor erreicht wurden, aber auch ältere Menschen in der Lage waren, die digitalen Angebote zu nutzen (vgl. Wehrstein und Zettl 2020, S. 7). In den offenen Antworten wurden zudem besondere Gruppen genannt, die neu erreicht werden konnten, unter anderem Eltern, Schichtarbeiter*innen und Kranke (vgl. ebd., S. 13).

Die Follow-Up-Befragung des midi-Instituts zeigt schließlich, dass der Anteil der digital angebotenen Gottesdienste im Laufe der Corona-Pandemie selbst im infektionsärmeren Sommer nur geringfügig zurückgeht. Im zweiten „Lockdown“ des Winters 20/21 übersteigt der Wert mit 83 % sogar den Ursprungswert aus der ersten Zeit der Kontaktbeschränkungen (vgl. Hörsch 2021, S. 24). Zeitgleich werden von den allermeisten Gemeinden auch wieder Gottesdienste vor Ort angeboten – von beinahe 100 % der Befragten nach dem ersten Lockdown, von immerhin knapp drei Viertel der Befragten auch während der zweiten „Lockdownwelle“ vom Winter 2020 bis Frühjahr 2021 (vgl. ebd., S. 23). Diese Gottesdienste wurden dann häufig zusätzlich aufgezeichnet und asynchron online zur Verfügung gestellt (vgl. ebd.). Sicher auch deshalb nahm die geschätzte Reichweite der digitalen Gottesdienste und Andachtsformate im Vergleich zur ersten Zeit der Kontaktbeschränkungen um etwa 50 % ab (vgl. ebd., S. 28). Das Angebot an digitalen Gottesdiensten blieb also über die Folgemonate annähernd stabil, die Teilnehmerzahlen allerdings sanken.

Die Gottesdienste, Andachten und andachtsähnlichen Formate, auf die die Midi-Studie fokussierte, wurden zunächst vor allem und etwa in gleichem Maße über YouTube und jeweils die eigene Webseite gestreamt. Im Vergleich zu Digitalformaten der prä-Corona-Zeit gewinnt damit vor allem YouTube an Zuwachs, während die Webseite an Bedeutung verliert (vgl. Hörsch 2020, S. 26). Im weiteren Verlauf der Pandemie nahm insbesondere die Nutzung der Plattform YouTube noch weiter zu und wurde in 99 % der erhobenen Fälle für die Bereitstellung digitaler Angebote genutzt. Einen Zuwachs, wenn auch auf deutlich niedrigerem Niveau, erlebten auch Videokonferenztools wie Zoom oder Microsoft Teams, außerdem Facebook, Instagram und Messengerformate (vgl. Hörsch 2021, S. 27). Hinsichtlich der Ausgestaltung der Angebote gaben in der ersten Erhebung 60 % der Befragten an, dass keine interaktive Beteiligung möglich war, in den anderen Fällen wurden als Beteiligungsoptionen überwiegend die Gelegenheit zum Mitbeten und Mitsingen sowie zum anschließenden Feedback gegeben (vgl. Hörsch 2020, S. 34). Für social media typische Optionen zur Interaktivität wie etwa ein Live-Chat wurden nur in etwa einem Viertel der Fälle angeboten (vgl. ebd., S. 35).

Während die Midi-Studie evangelische Gemeinden in den Blick nimmt und die Digitalstudie zwar überkonfessionell angelegt ist, aber den größten Teil ihrer Antworten doch im evangelisch-landeskirchlichen und evangelisch-freikirchlichen Feld generiert, ist die Contoc-Studie konfessionsübergreifend angelegt und umfasst gleichermaßen das katholische Feld. Sie fokussiert vor allem den Umgang von Hauptamtlichen der katholischen und evangelischen Kirche mit Digitalisierungsprozessen, aus Deutschland liegen mehr als 3000 Datensätze vor (vgl. CONTOC 2021, S. 3). Die Erhebung fand hier von Mai bis Juli 2020 statt, die Ergebnisse geben also auch Aufschluss spezifisch über die erste Pandemiephase, die durch Kontaktbeschränkungen und den Ausfall lokaler Gottesdienste gekennzeichnet war. Die Befunde der Studie sind zum Zeitpunkt der Abfassung dieses Aufsatzes noch kaum publiziert, die einsehbaren Daten legen aber nahe, dass sich das evangelische und das katholische Feld in manchen basalen Punkten kaum signifikant unterscheiden. So wurden etwa in beiden Traditionen während der Pandemie in sehr gleichem Maße digitale Angebote bereitgestellt, und auch die Einschätzung der Risiken und Chancen ebenso wie des Handlungsbedarfes hinsichtlich digitaler Angebote weist kaum Unterschiede auf (vgl. etwa CONTOC 2021, Abb. 1 und 2). Unterschiede zeigen sich in den schon einsehbaren Daten dort, wo sie mit innertheologischer Diversität einhergehen, etwa hinsichtlich der Digitalisierung von Abendmahl bzw. Eucharistiefeier (vgl. ebd., S. 11). Für allgemeine Aussagen zur Mediennutzung im Zuge digitalisierter Verkündigungsformate ist also tentativ eine gewisse Übertragbarkeit der oben skizzierten Befunde auf den katholischen Bereich anzunehmen^15^. Dies gilt nicht dort, wo christliche Traditionen einschlägige theologische und kulturelle Unterschiede und dementsprechende Differenzen in der liturgischen bzw. rituellen Ausgestaltung der Verkündigungsformate aufweisen, etwa hinsichtlich der sehr unterschiedlich verlaufenden innerkirchlichen Debatten um die Ausgestaltung des Abendmahls (vgl. einführend Deeg 2021b; Hirsch-Hüffell 2021).

## Die Rezipient*innenperspektive auf digitalisierte kirchliche Angebote

Die bisher genannten Studien haben ihre Datengrundlage in Erhebungen unter haupt- und ehrenamtlichen Beschäftigten in den christlichen Kirchen in Deutschland, die an der Digitalisierung gottesdienstlicher und weiterer Formate beteiligt waren oder diese verantwortet haben, und konnten so einen allgemeinen Überblick über die Digitalisierungsbemühungen christlicher Gemeinden in den ersten ein bis zwei Jahren der Pandemie geben. Aus religionswissenschaftlicher und -soziologischer Sicht ist weiterhin die Perspektive der Teilnehmer*innen an diesen Angeboten relevant. Sie kann den Angeboten und Intentionen dahinter erste Reaktionen und Rezeptionsmuster entgegenstellen und so den Blick auf die Gesamtverfasstheit dieses Feldes schärfen. Hier stellen sich zunächst explorierende Fragen nach der Annahme der Angebote, ihrem Stellenwert für die religiöse Praxis der Teilnehmenden, ihrem Blick auf Vorzüge, Nachteile und Irritationen der digitalisierten Version, nach sich entwickelnden und damit potenziell über die Corona-Krise hinausweisenden Vorlieben und sich neu etablierenden Routinen in individueller religiöser Praxis.

Als Datengrundlage kann hier vor allem auf zwei Studien zurückgegriffen werden: Die bereits kurz genannte „ReTeOg“-Studie in zwei Wellen 2020 und 2021 sowie eine erste, explorative Interviewstudie, die im eigenen Team durchgeführt wird.^16^ Die Interviewstudie besteht aus qualitativen Gruppeninterviews mit Teilnehmenden an digitalen Gottesdienstformaten. Die hier verwendeten Daten entstammen einer Gruppendiskussion mit sechs Teilnehmenden an den digitalen Gottesdiensten einer westdeutschen, katholischen Gemeinde.^17^ Die Gemeinde ist im urbanen Raum beheimatet (Katholik*innen und Protestant*innen machen hier je knapp 30 % der Bevölkerung aus), und lässt sich insgesamt als neuerungsfreudig beschreiben. Der Interviewleitfaden fragte nach den Erfahrungen in den Online-Gottesdiensten, nach Wünschen und Anliegen an die Online-Formate (insbesondere auch bezüglich der Interaktivitätsoptionen), nach Ritualisierungen der Teilnahme sowie nach Auswirkungen der Online-Erfahrungen auf die eigene Religiosität und die Erwartungen an religiöse Verkündigungsformate insgesamt. Die Daten wurden im Sommer 2021 erhoben und im Sinne der Grounded-Theory-Methodologie in einem dreistufigen Verfahren kodierend ausgewertet (vgl. Strauss und Corbin 1996). Die ReTeOg-Studie wurde in der ersten Erhebungswelle im Sommer 2020 durchgeführt und richtete sich an Mitglieder mehrerer evangelischer Landeskirchen, knapp 4800 Menschen haben teilgenommen (vgl. Arndt et al. 2020, S. 3). Auch die zweite Erhebung aus dem Sommer 2021 (Reimann und Sievert 2021) adressierte Mitglieder in den gleichen fünf EKD-Gliedkirchen, das Sample umfasst über 3800 Datensätze. Beide Erhebungen fokussieren spezifisch auf Online-Gottesdienste.

Von den Befragten in der ReTeOg-Studie, die sich durch die Art des Samplings gleichwohl durch einen gewissen selection bias auszeichnen, geben knapp 90 % an, Online-Gottesdienste besucht zu haben, und zwei Drittel, diese auch dann noch besucht zu haben, als wieder Präsenzgottesdienste stattfanden (vgl. Arndt et al. 2020, S. 6). Ungeachtet des vergleichsweise frühen Erhebungszeitpunktes im Frühsommer 2020 geben je ein Drittel der Befragten an, vier bis sechs Mal bzw. zehn Mal oder mehr an Online-Gottesdiensten teilgenommen zu haben (vgl. ebd., S. 8). In der zweiten Erhebungswelle 2021 hat der Anteil derjenigen, die auch bei existierenden Präsenzangeboten an Online-Gottesdiensten teilnehmen, von 65 % auf 55 % abgenommen (vgl. Reimann und Sievert 2021, S. 8) – angesichts der nun vergangenen eineinviertel Jahre von Online-Partizipation möglicherweise ein geringerer Schwund als erwartbar. Sowohl 2020 als auch 2021 wünschen sich etwa 80 % der Befragten die Beibehaltung von Online-Gottesdiensten auch nach der Corona-Zeit, mit nur minimal abnehmender Tendenz (vgl. ebd., S. 12). Dies deckt sich mit den oben skizzierten Befunden der Anbieter*innen: Die Online-Gottesdienste werden in hoher Zahl beibehalten und dies entspricht auch dem ausdrücklichen Wunsch der Befragten, die Teilnahme daran sinkt leicht ab. Zu keinem Zeitpunkt wird aber bis dato auch nur annähernd zu einem präpandemischen Status Quo zurückgekehrt.

Mit gut 60 % der Befragten gibt der größte Teil an, nur reine Gottesdienstübertragungen oder -aufzeichnungen angesehen zu haben, dialogische oder interaktive Elemente im Digitalen haben ein Viertel der Befragten erlebt (vgl. Arndt et al. 2020, S. 10). Knapp die Hälfte der Befragten gibt an, dass ihnen eine reine Übertragung oder Aufzeichnung genügen, knapp 40 % wünschen sich grundlegende oder umfassende dialogische Elemente (vgl. ebd., S. 14). Dies betrifft im überwiegenden Teil der Fälle das Einbringen von Fürbitten, in geringerem Maße auch die Einbindung von Diskussionselementen und von interpersonellem Austausch vor oder nach dem Gottesdienst (vgl. ebd., S. 15). Auch zu diesem frühen Zeitpunkt ist das Bedürfnis nach stärkerer Interaktion also schon etwas größer ausgeprägt als es von den Anbietern bedient wird. In der Folgeerhebung 2021 hat der Anteil der Gottesdienste ohne jede Interaktion ebenso leicht zugenommen wie der der Gottesdienste mit umfassenden dialogischen Formaten – abgenommen haben solche Formate, die entweder nur grundlegende dialogische Elemente integriert haben oder aber ergänzendes Material vor Ort bereitgestellt haben (vgl. Reimann und Sievert 2021, S. 11). Dies lässt sich als Hinweis deuten, dass sich hier bestimmte Formate stärker etabliert haben und sich das Feld insgesamt etwas zurechtsortiert hat: Einerseits in gezielt dialogische, interaktive Formate, die den Teilnehmenden an ihren Bildschirmen den Austausch miteinander oder mit den Veranstaltenden erlauben^18^, andererseits in reine Aufzeichnungen bzw. Streamings lokaler Gottesdienste, die ohne weiteren Aufwand auf Videosharing-Plattformen wie YouTube zur Verfügung gestellt werden können. Gleichzeitig weist es noch einmal darauf hin, dass sich hinter den befragten Nutzer*innen eben durchaus unterschiedliche Typen von Teilnehmer*innen verbergen können, deren Nutzungsweisen und -bedarfe sich durchaus auch grundlegend voneinander unterscheiden können.

Die zweite Erhebungswelle der ReTeOg-Studie erlaubt außerdem einen genaueren Einblick in die religiösen Vorlieben und Bedürfnisse der Befragten in Bezug auf Online-Gottesdienste sowie die Veränderungen zwischen den zwei Erhebungswellen. Während sich in mancherlei Hinsicht wenig ändert – etwa in Bezug auf die gewünschte Dauer der Gottesdienste oder das verwendete Liedgut – zeigen andere Themenbereiche größere Veränderungen auf, vielleicht auch durch eine wachsende Vorstellungskraft, welche Variationen überhaupt denkbar sind. So nimmt etwa der Wunsch nach einer Studioecke im Kirchraum ab zugunsten einer Aufzeichnung unter freiem Himmel (vgl. ebd., S. 13). Besonders auffällig sind aber große Verschiebungen hinsichtlich des Faktors Interaktivität: Der Wunsch nach umfassender Interaktivität wird nun von über 20 % der Befragten geäußert (2020: 8,9 %), entsprechend hat der Anteil derjenigen abgenommen, denen zufolge eine reine Übertragung oder Aufzeichnung ausreicht (vgl. ebd., S. 13). Ähnlich gelagert sind auch die Plattformvorlieben: Von 25 % auf knapp 55 % mehr als verdoppelt hat sich der Wunsch nach Online-Gottesdiensten im Videokonferenz-Format, während andere präferierte Interaktionskanäle – soziale Netzwerke und Messenger, Email sowie der YouTube-Chat – deutlich an Boden verloren haben (vgl. ebd., S. 13). Es lässt sich vermuten, dass hier nun, vielleicht von erfolgreichen Beispielen angeregt, die ganzheitlichere Erfahrung der synchronen Videokonferenz (die auch zumeist einen Chat integriert) gegenüber aufgezeichneten Gottesdiensten mit zusätzlichen, aber asynchronen Austauschtools (wie Kommentarfunktionen) deutlich an Befürworter*innen gewonnen hat. All diese Daten können als Hinweise gelesen werden, dass zumindest für einen Teil der Befragten die Online-Gottesdienste von einer Notfalllösung zu einer viablen Alternative zum Präsenzgottesdienst geworden sind, die dann aber auch ihren Ansprüchen an interaktive Teilhabe und Emergenz von Gemeinschaftlichkeit entsprechen muss. Auch in der Digitalstudie, in der die Gelegenheit zu offenen Antworten gegeben wird, äußert sich explizit der Wunsch, nicht nur Streamingangebote bereitzustellen, sondern auch „Gemeinschaftsaspekte abzubilden“ und Interaktivität zu ermöglichen (vgl. Wehrstein und Zettl 2020, S. 12).

Diese Eindrücke können vertieft werden durch erste Befunde aus der bereits genannten, derzeit laufenden Interviewstudie mit Teilnehmer*innen an Gottesdiensten via Videokonferenzsoftware. Hinsichtlich der hier interessierenden Themen lassen sich aus ihnen mehrere Aspekte kondensieren, die teilweise Aufschluss geben über die Erfahrungen mit digitalen Angeboten im Allgemeinen, teilweise spezifisch sind für die medialen Konstellationen bei Videokonferenzsoftware.

Dass der Gottesdienst nicht im üblichen Kircheninnenraum abgehalten wird, bringt sehr augenfällige Veränderungen mit sich. Die Interviewten verweisen zunächst auf ganz pragmatische Defizite der lokalen Sakralarchitektur und heben Vorzüge des digitalen Tools hervor – etwa, dass man in den Kirchenräumen nicht von jedem Platz aus gut sehen kann und daher dort das Einbinden von Bilddokumenten schwierig oder unmöglich ist. Und während herkömmliche Musikbeiträge oder das gemeinsame Singen in der Online-Variante ungleich schwieriger zu realisieren sind, wird darauf verwiesen, dass die Akustik bei Wortbeiträgen gerade für schwerhörige Teilnehmer*innen in der heimischen Kirche häufig herausfordernd ist, nun am heimischen Rechner aber über die Lautstärkeregelung optimiert werden kann. Damit stellt der Online-Gottesdienst für einige Teilnehmergruppen sogar die barrierefreiere Variante dar. Andere Befragte äußern weitere Vorteile der Multimedialität via Videosharing-Software. Durch Verschriftlichungen (etwa der Namen Verstorbener, die sonst nur verlesen würden) oder die Einbindung von Bildwelten könnten manche Inhalte nun intensiver rezipiert werden:

„wo dann eben besondere Bildausschnitte, die manchmal kitschigromantisch und manchmal aber auch sehnsüchtig werden lassen, wenn man dann irgendwie auf so einem Steg am Strand oder irgendwie auf ein Waldgebiet guckt und dabei diesen Text hört, ist es noch einmal etwas anderes, als wenn man ihn eben im Kirchenraum, in der Gemeinde hört. Also das ist auch etwas, wo ich sage, das finde ich sehr ansprechend“ (Befragte 1)

Auch Videoimpulse sind in der Online-Version integrierbar und kommen „in aller extremen Brillanz in Bild und Ton auf dem Bildschirm zur Geltung“ (Befragter 2). Gleichzeitig ist die Online-Variante natürlich auch in anderer Hinsicht räumlich flexibel: Den Erzählungen der Befragten nach nehmen dort Menschen aus aller Welt teil, die beispielsweise vor einer Emigration Mitglied der Gemeinde waren, andere sind immobil und/oder temporär ans Krankenbett gefesselt. Insgesamt finden sich den Erzählungen zufolge Teilnehmende ein, die „sehr wahrscheinlich nicht einfach so im normalen Präsenzgottesdienst am Sonntag zu holen gewesen wären, da habe ich mich gefreut“ (Befragte 4). Die Online-Gottesdienste werden in der Konsequenz auch als potenzielle „Werbeveranstaltung“ (Befragte 1) verstanden, ermöglicht durch die Translokalität des Internets:

„Natürlich kann nicht jeder, der von irgendwie woher kommt, jetzt spontan in unsere Kirche kommen, wenn denn wieder Präsenzgottesdienst möglich ist. Aber vielleicht eben doch der ein oder andere, der sich zufällig dahin verirrt hat und doch aus [Ort] oder Umgebung kommt, sagt dann: Ja, das ist für mich ein Grund, auch häufiger noch einmal nach [Kirchengemeinde] zu gehen und eben doch über meinen Kirchenturm hinauszudenken“ (Befragte 1)

Damit sind zwei Aspekte von Räumlichkeit benannt, die im Falle der Online-Angebote die Teilnahme letztlich erleichtern: Der Wegfall der räumlichen Distanz, der neue Zielgruppen erschließen lässt und die Teilnahme für manche Gruppen erst ermöglicht, und der Wegfall baulicher Nachteile im Sakralraum bzw. die Möglichkeit, die digitale Alternative in der individuellen Nutzung auf die eigenen Bedürfnisse einstellen zu können. Insgesamt wird im Interview auch auf die hohe Teilnehmer*innenzahl verwiesen, die in dieser Gemeinde und zu diesem Zeitpunkt die üblichen Sonntagsgottesdienste deutlich übersteigt.

Die besonderen Eigenschaften von Zoom und ähnlichen Konferenztools reichen aber noch über die prinzipiellen Eigenschaften von Online-Angeboten hinaus. Sie erlauben nicht nur, räumliche Distanz zu verringern oder multimediale Komponenten einzubetten, sondern zeichnen sich eben auch durch die synchrone Anwesenheit aller Teilnehmer*innen aus. Im Gegensatz zu asynchronen Angeboten, aber auch Live-Streams via Videosharing-Plattformen können sich damit auch alle Teilnehmenden ins Gesicht sehen. Dies wird geschätzt von denjenigen, die so ihnen vertraute Gemeindemitglieder wiedersehen, aber auch von Gemeindefremden:

„die Fernsehgottesdienste hatte ich vorher verfolgt und fand die auch überwiegend sehr, sehr schön. […] aber irgendwie bin ich dann ein bisschen müde geworden dessen […] Und just in dem Moment stieß ich dann auf diesen online und das fand ich dermaßen persönlich. Also erst einmal die Menschen so nah … also das Gesicht so nah zu sehen von den Teilnehmenden oder von diesem Team.“ (Befragte 1)

Diese Wahrnehmung gilt übrigens auch im Vergleich zum analogen Gottesdienst: Nicht nur schaut man sich im Sakralraum in der Regel nicht in die Gesichter, sondern auf die Rücken und Hinterköpfe, verstärkt wird gerade in Zeiten des Maskentragens die Zoom-Variante als jene wahrgenommen, die es eher erlaubt, den anderen Teilnehmenden ins Gesicht zu schauen: „Wenn ich also in so einen Zoom-Gottesdienst reingehe, dann sehe ich deutlich mehr Gemeinde, als ich an anderer Stelle hätte sehen können“ (Befragter 2).

Damit ist bereits das Thema der Gemeinschaftlichkeit angesprochen, das sehr ambivalent betrachtet wird: Während einerseits viele Vorzüge gerade der synchronen Online-Gottesdienste mit sichtbaren Teilnehmer*innen hervorgehoben werden, die durchaus als gute Voraussetzungen für das Entstehen einer Gottesdienstgemeinschaft gelten können, ist das Empfinden von Gemeinschaft der Punkt, an dem dennoch die größten Desiderate benannt werden: „Wir haben jetzt viel darüber gesprochen, was der Zoom-Gottesdienst alles kann. Was ich … ein wichtiges Ding, was er nicht kann, ist die Gemeinschaft“ (Befragter 1). Konkreter wird dabei auf die fehlende Körperlichkeit verwiesen, die Berührungen, das in-den-Arm-nehmen oder auf-die-Schulter-klopfen. Diese gelten manchen der Befragten als notwendige Bedingungen für die situative Manifestation einer Gemeinschaft und sind online eben nicht realisierbar; anderen hingegen fehlen sie auch nicht.

All diese Veränderungen in der medialen Konstellation im Vergleich zum Offline-Äquivalent erzeugen Verschiebungen für die religiöse Praxis, die in den Erfahrungen der befragten Rezipient*innen jeweils recht konkrete Vor- oder Nachteile mit sich bringen. Es gibt allerdings noch einige weitere Punkte, die sich eher aus der Beobachtung einer tiefergehenden Anregung von Innovation und Freisetzung in der religiösen Praxis entfalten lassen. Einige Erzählungen in der Gruppendiskussion berühren etwa die individuelle Ausgestaltung der Teilnahme an den Online-Gottesdiensten. Die Befragten berichten hier, wie sie verschiedene eigene Routinen um die Online-Gottesdienste entwickeln. Zum Einen tangiert das noch einmal den Punkt der Gottesdienstgemeinschaft, insofern sich auf einmal neue Zuschauergemeinschaften bilden:

„Und gleichzeitig habe ich von Anfang an meine Oma mit eingeladen gehabt, weil die ja auch nicht in die Kirche gehen konnte und davon sehr betroffen war. Und dann haben wir uns eingerichtet, bei ihr den Gottesdienst zu gucken. […] Ich komme eine halbe Stunde eher, wir können noch einmal miteinander quatschen. Wir setzen uns dann zusammen vor den Computer. Wir nehmen an diesem Gottesdienst teil. […] was ich jetzt schon wieder fast vermisse, wo die Präsenzgottesdienste wieder loslegen und meine Oma in ihre eigene Kirche geht und ich eben woandershin.“ (Befragte 1)

Innerhalb dieser Zuschauergemeinschaften vor dem Bildschirm findet zudem ein Austausch statt, der in den traditionellen Gottesdiensten so nicht so einfach zu integrieren ist. Die ersten Unterhaltungen über den Sinngehalt der Predigt erfolgen teilweise direkt während der musikalischen Beiträge, aber auch die anschließende Diskussion der Inhalte, die durch das nahtlose Sitzenbleiben in der familiären Konstellation und auf dem heimischen Sofa befördert wird, intensiviert sich: „Und danach haben wir das oft genug noch einmal eine halbe Stunde, Dreiviertelstunde nachwirken lassen und alles diskutiert, was besonders war und was toll war und was irgendwie hängen bleibt“ (Befragte 1).

Die Interviewpartner*innen berichten weiterhin, in welcher Familienkonstellation sie den Gottesdienst schauen, wann sie sich einfinden, wie diese Abläufe ihren Sonntag verändern („Ich weiß gar nicht, wie ich das früher geschafft habe, schon früh irgendwie in die Kirche zu kommen mit allen Mann“, Befragte 2), und wie sie eben auch das Davor und das Danach des Online-Gottesdienstes gezielt gestalten („Also so bewusst, wie man sich auf den Weg zur Kirche gemacht hat, haben wir uns dann bewusst auf den Weg zum Bildschirm gemacht“, Befragter 2). Auch die Gestaltung der Gottesdienstteilnahme selbst wird durch die spezielle Konstellation der Online-Gottesdienste in Teilen den Teilnehmenden übertragen. So berichten die Gesprächspartner*innen teilweise über sich selbst, teilweise von den Blicken auf andere Bildschirme, dass nun beispielsweise zuhause individuell eine Kerze entzündet wird. Solche Beispiele machen sichtbar, wie im Falle der translokalen digitalen Gottesdienste die religiöse Praxis stärker als zuvor in die Hände der einzelnen Teilnehmenden übergeht. Sie zeigen damit auch, wie die religiöse Praxis in der Ausgestaltung gewissermaßen übergreift – vom Geschehen auf dem Bildschirm hinein in die private Umgebung der einzelnen Teilnehmenden – und sich so zudem individuelle Variationen entwickeln.

Solche Selbstermächtigung der Teilnehmenden zeigt sich nicht nur in der individualisierten Ausgestaltung des häuslichen Rahmens, sondern auch hinsichtlich der Interaktion im digitalen Gottesdienstsetting. Das lässt sich etwa am Beispiel der Fürbitten illustrieren: Gleichwohl auch im lokalen Gottesdienst der betreffenden Gemeinde prinzipiell die Gelegenheit besteht, Fürbitten einzubringen, ist die Hemmschwelle „das jetzt in dem Kirchenraum zu sagen“ (Befragter 1) offenbar deutlich höher. Online hingegen, aus „einer gewissen Privacy am Rechner“ (ebd.) heraus, scheint es, so berichten die Interviewpartner*innen, vielen Teilnehmenden leichter zu fallen, sich zu artikulieren. Können die Fürbitten beispielsweise in den Zoom-internen Chat geschrieben werden, kann mithin kurz über die Formulierung nachgedacht werden, „dann fließen die Buchstaben in einer beeindruckenden Vielzahl“ (Befragter 2). Der heimische Raum resultiert so in einem größeren „safe-space“-Empfinden, und die Teilnehmenden bringen sich auf diese Weise aktiver ein als in vergleichbaren Situationen im lokalen Gottesdienst. Der Zoom-Gottesdienst wird, was die Teilnehmenden betrifft, in diesem Untersuchungsfall damit letztlich zum interaktionsstärkeren Setting.

Das alles wiederum steht im Kontext weiterer Freisetzungen. Im Gegensatz zu Gottesdienstdigitalisierungen, bei denen die traditionellen Abläufe mehr oder weniger unverändert aufgezeichnet und ins Internet geladen werden, haben die Verantwortlichen der bisher untersuchten Gemeinde das Format insgesamt verändert, sowohl in Bezug auf das räumliche Setting als auch hinsichtlich der eingebundenen Gesprächsformate und Sprecher*innen. Innovationen wie das Einladen externer Gäste werden von denjenigen, die sich bereit erklärt haben, an den Gruppeninterviews teilzunehmen, positiv hervorgehoben: „war […] absolut nicht der zwanghafte Versuch, Kirche auf Bildschirmen nachzuspielen. Das hat die evangelische Kirche [eines anderen Ortes] gemacht und ist damit fürchterlich gescheitert“ (Befragter 2), und „ich schätze es an den Zoom-Gottesdiensten, dass sie eben so ganz anders sind, als wenn wir uns in der Kirche treffen“ (Befragte 2). Insbesondere gilt dies aber auch für die Stärkung der Stimmen der Lai*innen und Ehrenamtlichen, die nun stärker in die Gottesdienstgestaltung eingebunden werden. Auch die Frage, ob auch nicht-Geweihte predigen dürfen, wird zum Zeitpunkt des Interviews, so erzählen die Befragten, neu verhandelt. Die durch Covid19 erzwungene temporäre Ablösung vom Sakralraum und der Wechsel des Mediums mag also in diesem Fall auch breitere Freisetzungstendenzen angestoßen haben, die über die dem Medium geschuldeten, notwendigen Veränderungen hinaus gehen und bisherige Routinen in Frage stellen können – sei es, weil es nun einmal ohnehin einen Anlass dafür gab, Vertrautes auf den Prüfstand zu stellen, sei es, weil die Online-Gottesdienste auch in den kirchlichen Strukturen als Experimentierfeld gelten, in denen zumindest in der pandemischen Ausnahmesituation auch nicht jedes Jota der vorgabengetreuen Umsetzung unter die Lupe genommen wurde.

## Religion und digitale Medien – theoretische und systematisierende Anschlussstellen

Die konkreten empirischen Befunde der ersten nun vorliegenden Studien haben bisher für sich gestanden, nun sollen Perspektiven eröffnet werden, wie dieses noch junge Forschungsfeld an bestehende theoretische Debatten angeschlossen werden und so in weiterführende Überlegungen und Fragestellungen überführt werden kann. Dabei ist eine Vielzahl theoretisch-systematisierender Perspektivierungen denkbar, im Folgenden sollen mit dem Blick auf religiöse Gemeinschaftlichkeit, religiöse Praxis und individuelle Religiosität sowie dem Wandel religiöser Autorität einige besonders naheliegende Anschlussstellen im Mittelpunkt stehen. Dem noch vorgeschoben ist die Kontextualisierung der empirischen Befunde durch bisherige systematisierende Ansätze zu Religion und Digitalisierung während der Corona-Pandemie.

### Religion, digitale Medien und Corona: *Transferring, translating & transforming*

Dieser Aufsatz entsteht im Sommer 2022, damit sind gerade zwei Jahre seit Beginn der Coronapandemie vergangen und die publizierte wissenschaftliche Auseinandersetzung steht naturgemäß noch am Beginn. Die ersten Daten insbesondere zum Feld christlicher Kirchen und Gemeinden in Deutschland wurden hier bereits vorgestellt, in der deutschsprachigen Diskussion liegen ansonsten vor allem die eingangs genannten theologischen Auseinandersetzungen zu liturgischen und ekklesiologischen Fragen in digitalen Gottesdiensten vor. Im internationalen Raum hingegen gibt es bereits einige wenige Publikationen, die erste Reflektionen und Beobachtungen rund um das Feld vereinen (vgl. Campbell 2020, 2021). Besonders viel Resonanz erfahren hat dabei Campbells Unterscheidung dreier Strategien, mittels derer digitalisierte Kirche unter Corona umgesetzt wurde: *transferring, translating* und *transforming* (vgl. Campbell 2020, S. 51). „Transferring“ meint dabei, dass der übliche lokale Gottesdienst einfach in die Online-Umgebung übertragen wird, sei es durch das Hochladen auf eine Videosharing-Plattform oder durch das Livestreamen etwa via Facebook (vgl. ebd.). Dies kann mit oder ohne Teilnehmende vor Ort erfolgen, zentral ist, dass hier ein Gottesdienst angeboten wird, der den prä-Corona-Routinen so nahe wie möglich ist. Solch abgefilmte oder live gestreamte Gottesdienste ähneln mithin auch den bereits etablierten Fernsehgottesdiensten, außer, dass sie eben aus der eigenen Gemeinde gesendet werden sowie im Falle der Aufzeichnung jederzeit angeschaut werden können. Die zweite Strategie ist die des „translating“: Hier werden Gottesdienste und andere religiöse Praktiken so modifiziert, dass sie bildschirmtauglich werden – etwa durch die Aufzeichnung in einem eigens dafür eingerichteten Studio oder die Integration kleinerer interaktiver Elemente wie Kommentarspalten, in denen zu Feedback eingeladen wird (vgl. ebd.). Im Rückgriff auf die im letzten Kapitel ausgeführten empirischen Beispiele könnte zu diesem „translating“ auch die Nutzbarmachung weiterer medialer Möglichkeiten gehören, sofern sie im Sinne des ursprünglichen Formats sind – etwa, indem sie durch die Einbindung von Verschriftlichungen der verlesenen Texte oder eines Gebärdendolmetschers die Barrierefreiheit erweitern. Die dritte Strategie hingegen – „transforming“ – geht von einer ganz neuen Gestaltung kirchlicher Angebote aus, nämlich „to use the shift to online as an opportunity to rethink the essence of the church – what do members need – and transforming their worship services accordingly“ (ebd.). Campbell nennt beispielhaft ein Kamingespräch, in dem die Pastor*innen eben eine ganz andere Umgebung und auch Gesprächsführung wählen, um Themen rund um die Herausforderungen der Pandemie zu adressieren (vgl. ebd.).

Obwohl die Kategorien vielleicht nicht ganz trennscharf ausformuliert werden, geben sie dennoch eine gute gedankliche Strukturierungshilfe für dieses neue Feld an die Hand. Ein Großteil der Beispiele im deutschsprachigen Raum dürfte unter die ersten beiden Kategorien fallen. Radikalere Umgestaltungen, die in die Kategorie des „transforming“ fallen, finden sich deutlich seltener oder sie sind Teile von Mischformen – wie etwa die Zoom-Gottesdienste, auf die sich in den Gruppeninterviews bezogen wird und die einerseits ein vergleichsweise dialogisches Medium wählen, andererseits aber zentrale liturgische Teile beibehalten, und schließlich auch ganz unübliche Bestandteile wie Interviews mit wechselnden Gästen einbauen. Die vorgestellten Daten zeigen dabei, dass solch transformierte Formate zumindest von einer Gruppe der Kirchenmitglieder sehr geschätzt und geradezu als Hoffnungsschimmer für die Innovationskraft der christlichen Kirchen gedeutet werden. Zumindest in Bezug auf die Interaktivität erlaubt die Zusammenschau der verschiedenen Studien zudem die Diagnose, dass ein höheres (und weiter wachsendes) Interesse an interaktiven und dialogischen Elementen in den digitalen Gottesdiensten besteht als bisher adressiert wird. Gleichzeitig wird in den ersten Reflektionen deutlich, dass von Anbieterseite häufig auch einfach die Kompetenzen und Ressourcen fehlen, um solche Angebote zu realisieren (vgl. u. a. Campbell und Osteen 2021).

Solche Kategorisierungen wie jene von *transferring, translating* und *transforming* (ähnlich wie etwa die Unterscheidung von „religion online“ und „online religion“, die Helland (2000) in einer frühen Phase der religionswissenschaftlichen Erarbeitung des Forschungsfeldes von Religion und Internet prägte) zeigen gleichwohl gerade durch ihre Unschärfe vor allem die große Fluidität eines Feldes, das sich auch während seiner Erforschung in stetigem Wandel befindet – übrigens immer wieder auch angestoßen von der Forschung zum Feld selbst. Dass solche Kategorien sich damit stets nur annähern können, bis sie wieder verworfen werden, liegt daher in der Natur der Sache, sensibilisiert aber gleichzeitig, das ist ihr Vorzug, für notwendige Binnendifferenzierungen verschiedener medialer Kommunikationskonstellationen genauso wie für Spannungsverhältnisse zwischen Online-Erfordernissen und Offline-Traditionen. Für das Feld der hier beleuchteten Online-Gottesdienste im deutschsprachigen Raum machen sie auf diese Weise deutlich, dass der Umgang der christlichen Kirchen mit den Digitalisierungsherausforderungen häufig genug zwar in einem grundsätzlichen Transfer ins Digitale gemündet ist, so dass die oben zitierte Diagnose der Midi-Studie von einem „Digitalisierungsschub“ (Hörsch 2021, S. 21) zutrifft. Die vorgestellte Unterscheidung weist aber auch darauf hin, dass damit nicht gleichzeitig ein Innovationsschub impliziert ist, sondern sich hinter der Digitalisierung von Verkündigungsformaten auch ein inhaltlich wenig invasiver Medienwechsel verbergen kann, der nicht grundsätzlich mehr Transformationspotential als andere Verschiebungen der letzten Dekaden – wie etwa im Zuge der Einführung von Fernseh- und Radiogottesdiensten – aufbietet.

### Vergemeinschaftung online

Verschiedene systematische Perspektiven haben in den letzten Jahren das emergierende und facettenreiche Feld von Religion und digitalen Medien auf theoretische Foki hin verdichtet. Eine Handvoll von ihnen hat sich als so ergiebig und anschlussfähig erwiesen, dass sie immer wieder als Referenzpunkt für Analysen sowie zur Organisation des Forschungsstandes dienen. Insbesondere die religionswissenschaftlichen Kernkonzepte Gemeinschaft, Autorität und Identität sind immer wieder Kristallisationspunkte solcher Ordnungen, verschiedentlich außerdem theoretische Fassungen von Ritual, Körperlichkeit/Embodiment oder Authentizität (vgl. etwa Campbell und Tsuria 2022; Kołodziejska 2018; für eine Übersicht Campbell und Evolvi 2020). An die empirischen Beobachtungen in den vorangegangenen Abschnitten anschließend sollen nun einige Anschlusspunkte zu Gemeinschaft, Autorität und Ritual hergestellt und damit theoretische Ordnungen der empirischen Befunde vorgeschlagen werden. Dabei sind alle der genannten Felder eng miteinander verflochten, und insbesondere Fragen von Körperlichkeit sowie Raumbezügen entfalten ihre Einflüsse auch in allen anderen Themenbereichen.

Der Transfer religiöser Kommunikation und Praxis in digitale Medien hat immer wieder die Frage aufgeworfen, welche Sozialformen sich online bilden (können) und was dies für Konsequenzen für religiöse Gemeinschaft hat – umso mehr mit Blick auf religiöse Traditionen, für die Gemeinschaftlichkeit ein essentieller Bestandteil des religiösen Lebens ist. Abseits der frühen sowohl utopischen als auch dystopischen Deutungen des Internets auf seine Gemeinschaftspotentiale hin haben viele Studien detaillierte empirische Analysen vorgelegt dazu, wie sich Gemeinschaftlichkeit online formiert (vgl. u. a. Campbell 2005; Neumaier 2016; Hutchings 2017; Kołodziejska 2018). Dabei wird deutlich, dass ohne eine gründliche Binnendifferenzierung keine hilfreichen Ergebnisse zutage zu fördern sind: Erstens bieten unterschiedliche Medienplattformen unterschiedliche Opportunitätsstrukturen für das Entstehen religiöser Gemeinschaften, indem sie Kommunikation personalisierter oder anonymer, beständiger oder fluider, stärker top-down oder stärker als gleichberechtigen Austausch strukturieren. Zweitens und vorgeordnet stellt sich die Frage, was überhaupt als Gemeinschaft verstanden wird. Hier ist es letztlich der Rückgriff auf grundlegende soziologische Theorien (etwa Weber 2010; Tönnies 2005; Anderson 2006; Hepp 2008; Knoblauch 2008), der die entscheidenden Prämissen schafft für das Entdecken und Deuten von Gemeinschaftlichkeit online.

Die hier thematisierten Gottesdienste stellen eine eigentlich recht klar definierte Gattung religiösen Austausches dar. Sie werden aber im Transfer ins Digitale medial unterschiedlich umgesetzt: nicht nur, aber vor allem als Stream oder Upload auf Videosharingplattformen einerseits, als synchroner Gottesdienst via Videokonferenzsoftware andererseits. In diesen Varianten lassen sich unterschiedliche Ausgangsvoraussetzungen für das Entstehen von Gemeinschaftlichkeit identifizieren. Während in den o. g. breiteren, plattformunabhängigen Umfragen der Wunsch sichtbar wird, noch stärker soziale Interaktion zwischen Teilnehmenden und Veranstaltenden der Online-Formate zu ermöglichen, heben die Interviews mit Teilnehmenden an Zoom-Gottesdiensten entsprechend eben jene Eigenschaften der Plattform hervor, die in besonderer Weise den Aufbau von Sozialkontakten befördern. Insbesondere die gegenseitige Wahrnehmung in der synchronen Anwesenheit und der Möglichkeit, die anderen Teilnehmer*innen von Angesicht zu Angesicht zu sehen, kann ein Schlüssel zu einem stärkeren Gemeinschaftsgefühl sein. Dennoch bleibt der teilnehmerseitige Eindruck uneindeutig, denn einem Teil der Interviewpartner*innen gilt das Fehlen der körperlichen Berührung als entscheidender Mangel auf dem Weg zu einer vollständig empfundenen Gemeinschaft. Hier ist im Sinne mancher rezenterer Gemeinschaftsdefinitionen der soziologischen Theorie zu berücksichtigen, dass auch das eigene Empfinden, Teil einer Gemeinschaft zu sein, ein zentraler Bestandteil einer solchen Definition sein kann (vgl. etwa Anderson 2006). Nichtsdestotrotz kann aus den ersten Analysen beigefügt werden, dass in der Teilnahme an einem Online-Gottesdienst Gemeinschaft auch an anderer Stelle erfahren werden kann, nämlich in der Zuschauergemeinschaft zuhause vor dem Bildschirm, die sich in manchen der empirischen Fälle wiederum durch einen engeren Austausch als bei Offline-Gottesdiensten sowie eigene gemeinschaftliche Rituale auszeichnet. Die Teilnahme an den Online-Formaten ruft damit Gemeinschaft auf mindestens zwei Ebenen auf: Zuhause vor dem Bildschirm und im übertragenen Geschehen auf dem Bildschirm. Beides muss in zukünftigen Studien nicht nur für sich genommen untersucht werden, es entfaltet auch in gerade der Verbindung aus mehreren potenziell zeitgleich erfahrenen Gemeinschaften besondere Komplexität – die schließlich noch einmal überlagert wird durch die Deutungen, die jede*r Beteiligte individuell an die Situation heranträgt.

### Religiöse Praxis, Raum und Ritual

Der Blick auf Ritualpraxis in digitaler Religion hatte sein großes Momentum in der religionswissenschaftlichen Forschung fraglos mit dem Aufkommen der virtuellen Umgebung „Second Life“, die um 2010 für einige Jahre besonders populär war und Nutzerzahlen im zweistelligen Millionenbereich aufwies. In dieser „virtuellen Welt“ siedelten sich damals auch religiöse Gemeinschaften an, wurde religiöse Praxis – und nicht zuletzt auch Gottesdienste – vollzogen. Eine Reihe religionswissenschaftlicher Analysen haben dies zum Gegenstand einer relecture des Ritualbegriffes gemacht (vgl. u. a. Miczek 2008; Radde-Antweiler 2006, 2008). „Second Life“ existiert zwar noch, hat aber deutlich an Popularität verloren und ist dabei zudem nicht durch ähnliche digitale Welten, sondern vielmehr durch die sozialen Medien als primäre Plattformen für Interaktion und Kommunikation abgelöst worden. Damit ist auch die Frage nach dem Transfer religiöser Rituale in digitale Umgebungen in der religionswissenschaftlichen Forschung wieder in den Hintergrund gerückt. Angesichts der massenhaften Digitalisierung religiöser Praxis während der Corona-Pandemie verdient sie allerdings ein Comeback – wenn auch in einem ganz anderen Rahmen. Die Gottesdienste via Zoom und ähnlicher Plattformen können dabei gerade als etwas unaufgeregtere Variante des Medienwandels den Blick öffnen dafür, wie auch kleinere mediale Verschiebungen innerhalb von Medien, die uns aus täglicher Nutzung vertraut sind, Effekte in der religiösen Praxis zeitigen.

Greift man zurück auf die religionswissenschaftlichen Ausarbeitungen rund um rezenten Ritualtransfer, ist zunächst anzuerkennen, dass Rituale, aus der wissenschaftlichen Außenperspektive betrachtet, nie statisch existieren, sondern immer einer gewissen Dynamik unterworfen sind, weil sie naturgemäß Teil eines kulturellen und gesellschaftlichen Kontextes sind, der sich wandelt (vgl. Lüddeckens et al. 2006, S. 1). Dies gilt dementsprechend auch für den Transfer von Ritualen in den Online-Kontext: „the way a ritual is presented, the way the rules are developed or changed, how people engage with the activity … The online environment challenges all of these aspects“ (Helland und Kienzl 2022, S. 43). Vergleichbar zur bereits zitierten Unterscheidung Hellands in „religion online“ und „online religion“ (vgl. Helland 2000) ist für das Feld der Rituale die Unterscheidung von „ritual online“ und „online ritual“ (vgl. Miczek 2008) vorgeschlagen worden. Im Gegensatz zu online neu entstehenden Ritualen („online ritual“) handelt es sich bei den Online-Gottesdiensten dann um „rituals online“, also um die Übertragung eines offline bereits etablierten Rituals ins Digitale. Wie bei Hellands Unterscheidung auch regt aber die mediale Weiterentwicklung seit der Entstehung dieser Kategorie einerseits, die Beobachtung des Online-Gottesdienstes als konkretem Fallbeispiel andererseits in besonderem Maße dazu an, Rückfragen an die Kategorisierung selbst zu stellen: Wie das hier vorgestellte Datenmaterial andeutet, verbindet sich das transferierte „ritual online“ ja mit einer Eigenentwicklung eines „offline rituals“ – etwa dem Anzünden einer Kerze oder einer anderweitig einbettenden Gestaltung der Gottesdienstteilnahme – die zwar offline neu, aber gezielt und eigenständig in Bezug auf das Ritual online entwickelt wird. Oder, um es weniger akteurszentriert zu formulieren: Das Beispiel ragt möglicherweise über die Unterscheidung von „internen“ und „externen Dimensionen“ eines Rituals (vgl. Lüddeckens et al. 2006) hinaus, und fügt dem eine Verdopplung oder Vervielfältigung von Ritualebenen hinzu, indem das Ritual zeitgleich in zwei (aus der Sicht eines Teilnehmenden) oder zig (aus der Sicht der Veranstaltenden) translokal verstreuten Räumen stattfindet.

Die Erfahrung von Gemeinschaft ist für einige Teilnehmende mit der Erfahrung von körperlicher Ko-Präsenz verknüpft, und das dürfte ebenso für die Erfahrung von Räumlichkeit gelten. Der Körper ist dabei im Zuge der Covid19-Pandemie ein besonderer systematischer Kristallisationspunkt, schließlich bekommt er in der Verschränkung von Religion und Pandemie mindestens eine doppelte Signifikanz zugewiesen. Einerseits ist er im Kontext der Pandemie potenziell Gefährder und gefährdet. Die Verlagerung von religiösen Ritualen ins Digitale schließt daran an, das Unterbinden von Köperkontakt soll Ansteckungen vermeiden, und war auch nach Wiederaufnahme analoger Gottesdienste noch Gegenstand anhaltender Hygienemaßnahmen (vgl. u. a. Bawidamann et al. 2020, S. 8 f.). Gleichzeitig gilt das körperliche Erfahren eines Sakralraumes, der verschiedenen materiellen und sinnlichen Ebenen eines religiösen Rituals sowie eben die auch durch körperliche Nähe induzierte Gemeinschaftserfahrung häufig als essentieller Teil der Ritualpraxis (vgl. ausführlicher ebd., S. 14 f). Digitalisierung radiert körperliche Erfahrung einer Ritualpraxis nicht aus, aber verändert sie entscheidend – insbesondere, indem sie die sinnlichen Erfahrungen jeglicher normierten Einheitlichkeit durch gleiche externe Einflüsse in einem geteilten Raum entziehen. Die sinnlichen Wahrnehmungen in der Teilnahme an digitalen Verkündigungsformaten werden damit hochindividualisiert und haben ihren Ausgangspunkt wohl zumeist in einer Sphäre des Alltäglichen, wenn diese auch gezielt für die Teilnahme modizifiert werden mag. Damit verweist der Körper auf die Bedeutung von Materialität, Sinnlichkeit und Atmosphäre, und damit auf Aspekte, die in diesem Kontext einer weiterführenden Betrachtung lohnen (vgl. innerhalb der Theologien u. a. einführend Deeg 2019).

Die zeitgleich mehrebige Erfahrung von Gemeinschaft gilt im Fall der Online-Gottesdienste also auch für eine verschachtelt-doppelte Raumerfahrung: Vor dem Bildschirm sitzend, wird gleichzeitig der Raum der direkten Umgebung – das Wohnzimmer, das Schlafzimmer, vielleicht das Café – und der auf dem Bildschirm visualisierte Raum erfahren. Nicht zuletzt ethnografische Forschungen, die auf den digitalen Raum hin adjustiert werden (vgl. Boellstorff et al. 2012; Hine 2015; Neumaier 2021) könnten hier weiteren Aufschluss geben, wie diese Verschaltung unterschiedlicher Handlungs- und Erfahrungsräume von Ritual von den unterschiedlichen Beteiligten erfahren, gedeutet und gestaltet werden.

### Autorität

Diese Verdoppelung der Raumerfahrung, der Körpererfahrung und der Gemeinschaftserfahrung in den privaten Raum hinein bedeutet gleichermaßen, dass die Teilnehmenden an den Online-Gottesdiensten einen Gutteil der Ausgestaltung des Geschehens in die eigene Zuständigkeit überantwortet bekommen. Mit wem zusammen sie teilnehmen, in welchem räumlichen Setting, welche rituellen Praktiken sie rund um die Teilnahme entwickeln, obliegt ihnen und möglicherweise ihrer (meist familiär geprägten) Zuschauergemeinschaft. Es liegt damit schnell auf der Hand, dass diese Verschiebung ins Digitale auch Konsequenzen für den Wandel religiöser Autorität hat.

Der Wandel religiöser Autorität ist wohl eines der meistdiskutierten übergeordneten Themen in Bezug auf digitale Religion (vgl. u. a. Campbell 2007, 2010b; Turner 2007; Cheong et al. 2011; Possamai und Turner 2012; Hoover 2016). Dieses Forschungsfeld hat seinen Vorlauf bereits im Aufkommen der Massenmedien, bekommt aber noch einmal eine neue Dynamik mit dem Aufkommen des World Wide Web und insbesondere des „Web 2.0“, das verstärkt Interaktivität und nutzergenerierte Inhalte erlaubt. In der Regel lassen sich als Fragerichtungen empirischer Studien unterscheiden, inwiefern dabei a) etablierte religiöse Autoritäten in Frage gestellt werden, b) sich neue religiöse Autoritäten etablieren, c) neue Wege, Autorität zu erlangen, entstehen – und schließlich auch d) bestehende religiöse Autoritäten auf neuen Wegen neue Geltung erhalten. Radde-Antweiler und Grünenthal (2018) identifizieren dazu zwei Hauptperspektiven: Die erste gehe von einer Destabilisierung traditioneller religiöser Autorität aus (vgl. u. a. Hjarvard 2008), während die zweite vielmehr eine Reifizierung und Konsolidierung der Strukturen etablierter religiöser Autorität durch neue Medien annehme (vgl. u. a. Barzilai-Nahon und Barzilai 2005). Zahlreiche Fallstudien (vgl. für einen ordnenden Überblick Cheong 2022) weisen im Detail doch oft widersprüchliche Befunde aus. Auch dies muss einerseits mit notwendigen Binnendifferenzierungen erklärt werden – zwischen verschiedenen Dekaden der Medienentwicklung, verschiedenen Medienplattformen mit unterschiedlichen kommunikationsprägenden Eigenschaften und nicht zuletzt ganz verschiedenen religiösen Offline-Bezugskontexten. Wie im Falle von Gemeinschaft ist aber auch das Konzept von Autorität, auf das in den Studien Bezug genommen wird, unterschiedlich, und reicht von der impliziten Bezugnahme auf traditionelle Formen religiöser Autorität (wie Priester oder Imame, vgl. etwa Bunt 2018) über den Rückgriff auf allgemeine soziologische Konzepte von Autorität oder Herrschaft (etwa Lincoln 1994) hin zu Neuentwicklungen speziell auf das Feld digitaler Religion bezogen (vgl. Campbell 2007, 2010b; Radde-Antweiler und Grünenthal 2018).

In Bezug auf das hier diskutierte Feld zeigt sich zunächst erneut die Varianz der beiden Beispiele von aufgezeichneten oder live gestreamten Gottesdiensten via YouTube u. ä. einerseits, den Varianten via Zoom und anderen Videokonferenzplattformen andererseits: Während erstere die Teilnehmenden zu in der Regel unsichtbaren Zuschauer*innen machen, eröffnet die Videokonferenz je nach Einstellung einen interaktiven Raum, der die Teilnehmenden stärker zu sichtbaren Bestandteilen des Geschehens werden lässt. Die Selbstermächtigung zur Gestaltung der Gottesdienstfeier allerdings, die bereits im vorigen Abschnitt angesprochen wurde, zeigt eine potenzielle Aneignung religiös-gestalterischer Autorität in der Ritualpraxis, die nicht auf die Zoom-Variante beschränkt ist. Vielmehr kann sie genauso auch ein Weg aus der reinen Zuschauerrolle etwa beim Ansehen vorab aufgezeichneter Gottesdienste online oder im TV sein, und ergibt sich zunächst einmal nur aus dem Umstand, dass die Teilnehmenden die Gottesdienste in einer privaten Umgebung, die ihrer Gestaltungsmacht unterliegt, vollziehen.

Von den Fallbeispielen der vorangegangenen Kapitel ausgehend erscheint aber noch ein weiterer Aspekt relevant, nämlich, inwiefern der Druck zur Entwicklung alternativer Gottesdienstangebote in der Corona-Situation auch auf anderen Ebenen die Freisetzung aus üblichen Routinen befördert hat. Die Teilnahme an einem Zoom-Gottesdienst ist dann gar nicht entscheidend dafür, wie sehr die Teilnehmer dort bestehende religiöse Autoritätsstrukturen in Frage stellen können, aber umgekehrt möglicherweise ein Indikator dafür, dass sich seitens der Verantwortlichen in der spezifischen Gemeinde gezielt für einen unkonventionelleren Weg entschieden wurde. Greift man noch einmal auf die oben eingeführte Unterscheidung von „transferring, translating, transforming“ zurück, kann, allgemeiner gesprochen, der Grad des „transforming“ auch ein Gradmesser für eine Auseinandersetzung mit bestehenden religiösen Rollen und Autoritätsstrukturen sein. Das darf dabei allerdings nicht zu monokausal gedacht werden, um nicht die Komplexität denkbarer Autoritätskonzepte zu verkennen: Während von Interviewpartner*innen beschrieben wird, dass in den von ihnen besuchten Zoom-Gottesdiensten die Rolle religiöser Expert*innen geschwächt, eine niedrigschwellige Beteiligung Vieler ermöglicht und die Handlungsmacht der Lai*innen gestärkt wird, können auch bei den in medialer Hinsicht unkonventionellsten Formaten – etwa Instagram-Stories mit Verkündigungsinhalten – durchaus starke Formen von religiöser Autorität etabliert werden. Zahlreiche Beispiele religiöser Influencer*innen (vgl. Krain und Mößle 2020; Neumaier 2022) machen deutlich, dass mediale Innovation bei weitem nicht immer mit der Loslösung von eindeutigen Autoritätsstrukturen einhergehen, sondern vielmehr eine klar organisierte Top-Down-Kommunikation, geringe Augenhöhe mit den Follower*innen und die inhaltliche Vermittlung rigider Vorgaben zu einer religiösen Lebensführung mit sich bringen können. Campbells Entwurf einer „religious social shaping of technology“ (Campbell 2010a) verdeutlicht ergänzend, dass mediale Charakteristika nicht allein ausschlaggebend sind, sondern die Aneignung von Medien auch von der Anbieterseite her gedacht werden kann: Sie wird dann durch die Haltung der Offline-Gemeinschaft bzw der zugehörigen religiösen Tradition zu digitalen Medien, aber auch zum Stellenwert religiöser Autorität oder religiöser Gemeinschaft vorstrukturiert und in das konkrete Handlungsportfolio eingepasst.

## Ausblick

Was lässt sich für ein Feld im Wandel zu diesem Zeitpunkt also notieren? Zunächst auf die Gottesdienste hin betrachtet: Christliche Verkündigungsformate wurden, wie die Angebote anderer Religionsgemeinschaften auch, unter Corona einem Medienwechsel unterzogen und infolgedessen häufig in digitalisierter Form angeboten. Einiges eint die verschiedenen Wege, die im Zuge dessen eingeschlagen wurden, an anderer Stelle zeigen sich deutliche Binnendifferenzen etwa zwischen synchronen und asynchronen oder interaktiven und nicht-interaktiven Angeboten. Aus medienforschender Sicht eignen sich die Online-Gottesdienste daher auch als Beispiel für Fallkontrastierungen, in denen die Auswirkungen bestimmter medialer Konstellationen etwa auf religiöse Praxis oder Erfahrung untersucht werden sollen.

Gleichzeitig weist das Fallbeispiel an verschiedenen Stellen über sich hinaus: Einerseits ermöglicht es einen Blick in die Aushandlungsprozesse der religiösen Expert*innen in den Haupt- und Ehrenämtern, die im Transfer in die neue mediale Umgebung die bisherigen Üblichkeiten neu auf den Prüfstand stellen. Dass sie teilweise die Gelegenheit nutzen, in dem nun entstandenen Freiraum sich freizuspielen von Vorgaben, die den geteilten Überzeugungen zuwiderlaufen, zeigt sich immer dann, wenn Transformationen sichtbar werden, die über die dem Medienwechsel geschuldeten und notwendigen Adjustierungen hinausgehen. Andererseits ermöglicht das Beispiel der Online-Gottesdienste eine vertiefte Auseinandersetzung mit den Bedürfnissen der Gottesdienst-Teilnehmenden, die häufig erst durch die Umstrukturierung angeregt wurden, über die eigenen Präferenzen in Bezug auf Verkündigungsformate – etwa hinsichtlich deren Interaktivität – nachzudenken.

Die Digitalisierung der Gottesdienstformate kann weiterhin beitragen zu den bereits intensiv diskutierten Fragen der Forschung im Feld von Religion und Medien, etwa zum Wandel von Gemeinschaft, von Autorität, von religiöser Identität. Sie bringt aber auch noch einmal Perspektiven neu bzw. zurück in die Aufmerksamkeit, die in der religionsbezogenen Forschung zu digitalen Medien etwas weniger präsent sind, nämlich jene, die nach der Bedeutung von Körperlichkeit, Materialität, Raumerfahrung und Ritualtransfer in den verdoppelten Räumen der translokalen Teilnahme an digitaler Verkündigung fragen. Und schließlich lassen sich all diese Erkenntnisse, die sich direkt aus der Empirie und ihrer systematisierenden Analyse ergeben, auch einspeisen in breitere religionssoziologische Debatten über gegenwärtige Religiosität: Sie geben dann etwa Hinweise auf ein zunehmendes Bedürfnis nach Interaktivität auch im Feld routinierter christlicher Gottesdienstbesucher*innen oder zeigen Konkretisierungen von Prozessen der Selbstermächtigung und Individualisierung religiöser Praxis. Damit können sie auch Tiefenschärfe bieten: Wenn etwa die Verlagerung zu Online-Gottesdiensten gleichzeitig Bedürfnisse nach Wahlfreiheit und Eigenständigkeit, aber auch nach religiöser Gemeinschaft aufzeigt, kann das auch die Dichotomisierung mancher Ansätze in Bezug auf den Wandel gegenwärtiger Religiosität mit Zwischentönen unterfüttern. In der forschenden Annäherung verweisen sie zuletzt erneut auf einen Forschungsgegenstand, der sich besonders fruchtbar triangulierend und idealerweise unter Einbeziehung digitalethnografischer Verfahren adressieren lässt.

Die vorliegende Erörterung muss naturgemäß einige blinde Flecken unadressiert lassen. Neben dem Blick in die binnentheologischen Debatten betrifft das auch die ausdifferenzierende Erörterung der unterschiedlichen Affordanzen verschiedener Medienplattformen und ihrer Konsequenzen, die unterschiedlichen Ressourcen und Herangehensweisen verschiedener religiöser Institutionen und ihrer Organisationsebenen, einen detaillierteren Blick auf verschiedene Nutzergruppen und deren Bedürfnisse auch in verschiedenen Pandemiephasen sowie zuletzt einige inhaltliche Vertiefungen, etwa zu Materialität, Atmosphäre und Körperlichkeit. Auch hier ergeben sich naheliegenderweise weitere Anschlussstellen für vertiefte empirische Forschung und theoretische Ordnung.

Wie sich konkret das Feld der Online-Gottesdienste entwickeln wird, zeigen die nächsten Jahre. Die Ressourcen kirchlicher Anbieter sind in dieser Hinsicht endlich und die Corona-Pandemie scheint derzeit nicht mehr zu verordneten oder selbstgewählten Kontaktbeschränkungen in nennenswerter Zahl zu führen, auch wenn man die Bedeutung vulnerabler Gruppen unter den Kirchenmitgliedern nicht unterschätzen darf. Gleichzeitig zeigt sich auch in den zurückliegenden Phasen der Pandemie, in denen ein vergleichsweise sicherer Gottesdienstbesuch möglich war – etwa den Sommern mit niedrigen Inzidenzwerten – ein anhaltendes Interesse an der Bereitstellung und auch der Teilnahme an Online-Gottesdiensten. Hier sind es häufig Gründe ganz jenseits der Pandemie, die die Online-Alternative attraktiv erscheinen lassen: das Erschließen neuer Zielgruppen, der translokale und barrierefreie Zugang, aber auch die Möglichkeit zur verstärkt eigenen Gestaltung der Teilnahme. Das legt nahe, dass die Online-Gottesdienste in einem gewissen Maße persistent bleiben werden. Als stetige Alternative, die sich dadurch auszeichnet, Offline-Routinen auf den Prüfstand zu stellen, wird sie damit ganz unabhängig von ihren Teilnehmenden auch ausstrahlen auf religiöse Offline-Angebote und die an sie gerichteten Erwartungen.

## Footnotes

1 Verlautbarung der Bundesregierung auf https://www.bundesregierung.de/breg-de/themen/coronavirus/besprechung-der-bundeskanzlerin-mit-den-regierungschefinnen-und-regierungschefs-der-laender-vom-22-03-2020-1733248, zuletzt abgerufen am 12.08.2022.

2 S. die Berichterstattung beispielsweise der Frankfurter Allgemeinen Zeitung, https://www.faz.net/aktuell/rhein-main/region-und-hessen/hunderte-muslime-beten-auf-ikea-parkplatz-in-wetzlar-16789071.html, zuletzt abgerufen am 12.08.2022.

3 Vgl. u. a. https://www.domradio.de/artikel/ostern-im-ausnahmezustand-corona-veraendert-2020-das-wichtigste-fest-der-christenheit, https://www.kirchetrais.de/2020/04/06/stationenweg-zur-passionszeit-ostern/, zuletzt abgerufen am 12.08.2022. S. hierzu grundlegend gliedernd auch Bawidamann et al. (2020), die die Modifikationen religiöser Rituale in Veränderungen (des Formats bei gleichbleibendem Ort und Zeit), Verlagerungen (der Örtlichkeit) und Absagen gliedern.

4 S. grundlegend u. a. Campbell (2013, 2017), Campbell und Evolvi (2020), Enstedt et al. (2015), Krüger (2012), Moberg und Sjö (2021).

5 Vgl. für einen Überblick mit verschiedenen Fallbeispielen Lüddeckens et al. (2006).

6 Vgl. in Bezug auf christliche Gottesdienste unter anderem Fischer (1985, 1987).

7 Tatsächlich stellten sich Gottesdienste nach Aufhebung der restriktiven Kontaktbeschränkungen für religiöse Kontexte vereinzelt auch als Ansteckungsort heraus, vgl. u. a. https://www.spiegel.de/panorama/gesellschaft/corona-behoerden-melden-65-infizierte-nach-gottesdienst-in-freikirche-a-f43005ee-59ae-45e2-bc3e-8e38f9943de9, zuletzt abgerufen am 12.08.2022.

8 S. die Projektwebseite https://contoc.org/de/contoc/, zuletzt abgerufen am 12.08.2022.

9 Siehe u. a. Schlag (2022).

10 S. https://contoc.org/de/contoc-2/, zuletzt abgerufen am 12.08.2022.

11 Auf die Unterschiede zwischen den Landeskirchen wird in der weiteren Darstellung der Befunde nicht eingegangen, diese können Hörsch (2020) entnommen werden.

12 Dabei kann hier nicht auf den Unterschied zwischen Landeskirchen bzw. Bistümern oder einzelnen Gemeinden eingegangen werden. Hörsch (2021) weist Unterschiede zwischen Landeskirchen gelegentlich aus, in CONTOC2 (2021) werden Unterschiede zwischen Katholik*innen und Protestant*innen aufgezeigt, in Contoc (2022) Länderunterschiede zwischen evangelischen Kirchen in Deutschland und der Schweiz. In anderen Datenauswertungen bleibt die Frage, inwiefern auch institutionelle Ressourcen oder Haltungen unterschiedliche Auswirkungen auf die Digitalisierung der Verkündigungsformate haben können, bisher weitgehend unberücksichtigt.

13 Gleiches gilt für digitale Angebote, die sich gezielt an Senior*innen richteten, besser sieht es aus bei zielgruppenspezifischen Angeboten in der Kinder- und Jugendarbeit (vgl. ebd., S. 40).

14 Auch in den ersten Auswertungen der CONTOC-Studie, wie sie von Wolfgang Beck im Frühjahr 2021 vorgestellt wurden, stimmen knapp 90 % der Befragten aus dem evangelischen und über 80 % der Befragten aus dem katholischen Feld der Aussage zumindest in Teilen zu, dass mit den Online-Gottesdiensten Menschen erreicht worden seien, zu denen sonst kein Kontakt bestanden habe.

15 In Bezug auf die Mediennutzung zeigt sich dies auch in Studien (vgl. MDG-Trendmonitor 2021, S. 98 f.), die nahelegen, dass sich beispielsweise katholische Befragte in ihrer Mediennutzung auch nur in Nuancen von der allgemeinen Bevölkerung unterscheiden.

16 Für die Planung und Durchführung der Gruppeninterviews danke ich Christopher Pilz.

17 Die Gruppe wurde dabei aus regelmäßigen Teilnehmer*innen der digitalen Gottesdienste, ansonsten aber möglichst divers zusammengesetzt. Sie umfasste vier Frauen und zwei Männer verschiedenen Alters, von denen zudem nicht alle Mitglieder der Gemeinde sind, sondern sich in einem Fall auch aus dem Ausland zuschalten.

18 Dies erfolgt häufig über den schriftlichen Austausch in einem parallellaufenden Chat, bei Gottesdiensten via Videosoftware auch durch den mündlichen Austausch etwa in ausgewählten Abschnitten des Gottesdienstes.
